# A Nutritional Approach for the Management of Chemotherapy-Induced Diarrhea in Patients with Colorectal Cancer

**DOI:** 10.3390/nu14091801

**Published:** 2022-04-26

**Authors:** Salvatore Artale, Nunziata Grillo, Stefano Lepori, Chiara Butti, Antonella Bovio, Sabrina Barzaghi, Andrea Colombo, Elena Castiglioni, Lucia Barbarini, Laura Zanlorenzi, Paola Antonelli, Riccardo Caccialanza, Paolo Pedrazzoli, Mauro Moroni, Sabrina Basciani, Rebecca Azzarello, Francesco Serra, Alessandra Trojani

**Affiliations:** 1 Oncology Unit, Gallarate-Busto Arsizio Hospital, ASST Valle Olona, 21013 Gallarate, Italy; ngrillo.nutrizione@gmail.com (N.G.); stefano.lepori@asst-valleolona.it (S.L.); chiara.butti@asst-valleolona.it (C.B.); antonella.bovio@asst-valleolona.it (A.B.); sabrina.barzaghi@asst-valleolona.it (S.B.); andrea.colombo@asst-valleolona.it (A.C.); castiglioni.elena@asst-valleolona.it (E.C.); lucia.barbarini@asst-valleolona.it (L.B.); laura.zanlorenzi@asst-valleolona.it (L.Z.); paola.antonelli@asst-valleolona.it (P.A.); 2 Clinical Nutrition and Dietetics Unit, Fondazione IRCCS Policlinico San Matteo, 27100 Pavia, Italy; r.caccialanza@smatteo.pv.it; 3 Oncology Unit, Fondazione IRCCS Policlinico San Matteo, Department of Internal Medicine and Medical Therapy, University of Pavia, 27100 Pavia, Italy; p.pedrazzoli@smatteo.pv.it (P.P.); francesco.serra01@universitadipavia.it (F.S.); 4 Oncology Division, San Carlo Borromeo Hospital, ASST Santi Paolo e Carlo, 20142 Milan, Italy; mauro.moroni@asst-santipaolocarlo.it; 5 Department of Experimental Medicine, Sapienza University of Rome, 00161 Rome, Italy; sabrinabasciani@yahoo.it; 6 Biology for Nutrition Sciences, University of Study of Milan, 20142 Milan, Italy; rebecca.azzarello23@outlook.it; 7 Department of Hematology, ASST Grande Ospedale Metropolitano Niguarda, 20142 Milan, Italy; alessandra.trojani@ospedaleniguarda.it

**Keywords:** cancer therapy-induced diarrhea, colorectal cancer, diet, Vitamin D

## Abstract

This study aimed to determine if dietary modifications using a nutritional regimen could prevent or reduce the incidence of cancer therapy-induced diarrhea in patients with metastatic colorectal cancer and to evaluate the relationship of Vitamin D blood levels with diarrhea severity. Patients with metastatic colorectal cancer were enrolled. A Mediterranean diet, containing some special limitations aiming to reduce the risk of diarrhea, was administered before and during the entire chemotherapy program. Enrolled patients numbering 60/137 (44%) had diarrhea during chemotherapy. Adherence to the diet was high in 36 (26.3%) patients, medium in 94 (68.6%), and low in 7 (5.1%). Mean adherence to the diet was significantly lower in patients who experienced diarrhea with maximum grade 2–3 compared to those who had no diarrhea or grade 1 diarrhea (score = 5.4 ± 1.9 vs. 7.1 ± 1.5, *p* < 0.001). Patients with higher adherence to the diet had a lower risk of grade 2–3 diarrhea (odds ratio: 0.5 (95% CI: 0.3–0.7, *p* < 0.001)). In addition, patients who completed a higher number of chemotherapy cycles had an increased risk of grade 2–3 diarrhea (odds ratio: 1.2 (95% CI: 1.0–1.5, *p* = 0.02)). Of note, a lower level of Vitamin D correlated with an increased risk of G2-G3 diarrhea (*p* = 0.03). A diet based on vegetables with a controlled fiber content, Mediterranean Modified Healthy Diet (MMHD), is useful to control the incidence of cancer therapy-induced diarrhea.

## 1. Introduction

Cancer therapy-induced diarrhea (CTID) is a frequent complication of chemotherapy and targeted therapy, occurring in more than half of the patients treated for colorectal cancer [1,2]. Chemotherapy with irinotecan alone and regimens, such as FOLFIRI (irinotecan + leucovorin + 5-fluorouracil), FOLFOX (oxaliplatin + leucovorin + 5-fluorouracil), and CAPOX (oxaliplatin + capecitabine), which are used for metastatic colorectal cancer, were reported to be associated with an increased incidence of grade 3–4 diarrhea ranging from 16% to 23% [3,4,5,6,7]. The loss of fluids and electrolytes associated with persistent or severe diarrhea can result in life-threatening dehydration, renal insufficiency, and electrolyte imbalances and may contribute to cardiovascular morbidity [8]. In addition, grade 3 or 4 diarrhea may impair therapy outcomes and increase mortality due to dose delays and reductions and/or interruption [9,10,11]. Finally, chemotherapy-induced diarrhea may persist many years after treatment, greatly reducing the quality of life [1]. Pharmacological and non-pharmacological treatments currently used, including loperamide, probiotics, or octreotide, are often ineffective and have little impact on outcomes [12].

Among nutritional requirements during treatment, patients receiving chemotherapy were found to have lower Vitamin D levels compared to patients receiving only surgical treatment; in addition, supplementation with Vitamin D analogs enhanced the sensitivity of colon cancer cells to 5-FU both in vitro and in vivo, suggesting an anticancer effect [13]. On the other hand, it is known that a Vitamin D deficiency may be associated with symptoms including loss of appetite, fatigue, diarrhea, and poor tolerability to chemotherapy [14,15].

Nutritional management of patients undergoing chemotherapy for colorectal cancer could reverse Vitamin D deficiency and facilitate the correction of electrolyte imbalance, limiting both the intensity and duration of diarrhea and promoting a re-alimentation process. With this approach, the prevention or amelioration of diarrhea may be expected to facilitate the regular completion of chemotherapy courses.

This study aimed to determine if dietary modifications using a nutritional regimen defined according to the World Cancer Research Fund (WCRF) prevention recommendations could prevent and/or reduce the incidence of CTID in patients with metastatic colorectal cancer and to evaluate the relationship between Vitamin D blood levels and diarrhea severity [16].

## 2. Patients and Methods

A prospective interventional study was performed after notification to the Ethics committee of Varese on 15 December 2015. The study was performed in accordance with the revised version of the declaration of Helsinki (52nd WMA General Assembly, Edinburgh, Scotland, October 2000). All patients provided their written consent to process personal data and to publish the study results. Recruitment was programmed within 18 months.

### 2.1. Patients

Consecutive patients with metastatic colorectal cancer, aged ≥ 18 years, with Eastern Cooperative Oncology Group performance status (ECOG PS) 0–2, and undergoing chemotherapy with fluoropyrimidine, oxaliplatin, and irinotecan (irinotecan alone, CAPOX, FOLFOX, or FOLFIRI regimens) were enrolled after patients provided written informed consent.

To avoid life-threatening toxicities, patients who were found to be homozygotes for DPYD*2A, *13, and rs67376798 were excluded, while in heterozygotes, a 50% dose reduction in chemotherapy was performed according to the Italian Association for Medical Oncology (AIOM) guidelines [17]. In addition, patients with cognitive disorders, metabolic disorders, concomitant radio-chemotherapy, ongoing radiotherapy, ECOG PS > 2, and homozygotes dihydropyrimidine dehydrogenase (*DYPD*) mutations were excluded.

### 2.2. Intervention

A diet was defined by a nutritionist based on a Mediterranean diet, with some special limitations aiming to reduce the risk of diarrhea (Mediterranean Modified Healthy Diet, MMHD). It was administered before and during the entire chemotherapy program. The diet was elaborated on by matching WCRF cancer prevention recommendations and based on the authors’ previous experience with neuroendocrine tumor patients [18]. WCRF recommendations included the following: be a healthy weight; be physically active; eat whole grains; vegetables; fruit and beans; limit fast food; limit red and processed meat; limit sugar-sweetened drinks; avoid alcohol consumption; do not use supplements for cancer prevention; breastfeed your baby; and after a cancer diagnosis, follow our recommendations, if you can [16]. A daily caloric intake of 1818 kcal was provided with a fiber intake of 22.90–30.36 g, corresponding to the minimum daily physiological amount of fiber = 12.6–16.7 g/1000 kcal [19]. Fibers were limited to minimize the induction of diarrhea. Whole grains were excluded to match the required amounts of fibers, while legumes, vegetables, and fruits were limited. To limit stool, daily vegetables portions were reduced; vegetable variety was limited [20]; sugar content was low and appropriately distributed during the day; and meals were small and frequent, at room temperature, and contained mainly dry foods [21,22]. Soft drinks were not admitted. Lactose was not included as 40% of the Italian population are intolerant, and a global prevalence of lactase non-persistence (LNP) phenotype is estimated at 68% [23,24]. The daily diet is reported in Supplementary Materials.

Supportive treatments: Diarrhea was managed according to guidelines for the treatment of CTID, with non-pharmacologic (probiotics) and pharmacologic (loperamide, octreotide) interventions [12,25,26]. Patients with grade 1–2 diarrhea without risk factors (moderate to severe cramping, grade ≥ 2 nausea/vomiting, decreased performance status, fever, sepsis, neutropenia, frank bleeding, or dehydration) were considered uncomplicated and managed conservatively with dietary modifications and loperamide. Patients with grade 3–4 diarrhea received probiotics, loperamide, octreotide, antibiotics, and parenteral nutrition. According to blood examinations, dehydrated patients were hospitalized for hydration and other supportive therapy.

To prevent nausea and vomiting, a 5-HT3 antagonist with corticosteroids was the standard premedication prior to infusion with oxaliplatin. For delayed nausea and vomiting, an oral 5-HT3 antagonist was administered, and metoclopramide, alizapride, and prochlorperazine could be added.

Hematopoietic growth factors (i.e., G-CSF or GM-CSF) were admitted and were administered according to current guidelines to treat febrile neutropenia [17].

### 2.3. Assessment

Diarrhea was defined as the passage of three or more loose or liquid stools per day (or more frequent passage than is normal for the individual) [27].

At baseline, demographic and clinical oncological data were recorded; blood examinations assessed *DYPD* mutations and Vitamin D levels (reference values were: sufficient = 30–100 ng/mL; insufficient = 10–30 ng/mL; deficient ≤ 10 ng/mL). Patients were screened for malnutrition and obesity using the five-step Malnutrition Universal Screening Tool (MUST) [28].

After each chemotherapy cycle, patients were evaluated for performance status (ECOG PS), diarrhea occurrence (named according to the National Cancer Institute’s (NCI) Common Terminology Criteria for Adverse Events (CTCAE) 4.0; events were recorded on a daily diary), adherence to diet (using a modified KIDMED test), diarrhea medical treatment, duration of toxicity, chemotherapy schedule modification, and impact on quality of life (using a numerical rating scale, NRS (from 0 = absence of impact to 10 = maximum impact)).

The KIDMED test is designed to evaluate the adherence to the Mediterranean diet of children and young people [29]. As the diet used in this study introduced several changes to the Mediterranean diet to limit the risk of diarrhea and excluded dairy foods, the KIDMED test was changed accordingly [18]. The PREDIMED test, which is validated for use in adults, did not meet our diet and was not used [30]. Adherence to the prescribed diet was considered high for scores ≥ 8, medium for scores 4–7, and poor for scores ≤ 3 [31].

### 2.4. Statistical Analysis

Based on the literature, the incidence of grade 3–4 post-chemotherapy diarrhea in metastatic colorectal cancer patients receiving irinotecan, FOLFIRI, FOLFOX, or CAPOX without nutritional intervention is around 20%, and an incidence of chemotherapy-induced diarrhea grade 3–4 ≤ 10% was considered a clinically relevant objective.

On this basis, the sample size was 137 subjects, with a target significance level of 0.05.

Data were analyzed with descriptive methods. Comparisons of groups were analyzed by ANOVA or Mann–Whitney tests. Univariate logistic regression was calculated to analyze if any dependent variable was associated with grade 2 or grade 3 diarrhea.

## 3. Results

Overall, 137 patients were enrolled; 63 (46%) were males, and the mean age was 65.3 ± 11.4 years. Demographic and clinical characteristics at baseline are reported in Table 1.

Overall, 60/137 (44%) patients had diarrhea during chemotherapy. within addition to chemotherapy cycles, the maximum grade of diarrhea was 1 in 42 (31%) patients, 2 in 12 (9%) patients, and 3 in 6 (4%) subjects. The mean score for adherence to the diet throughout chemotherapy cycles was 6.9 ± 1.7, with a median value of 7 ranging from 2 to 10. Adherence to the regimen was high in 36 (26.3%) patients, medium in 94 (68.6%), and low in 7 (5.1%).

**Figure 1:**
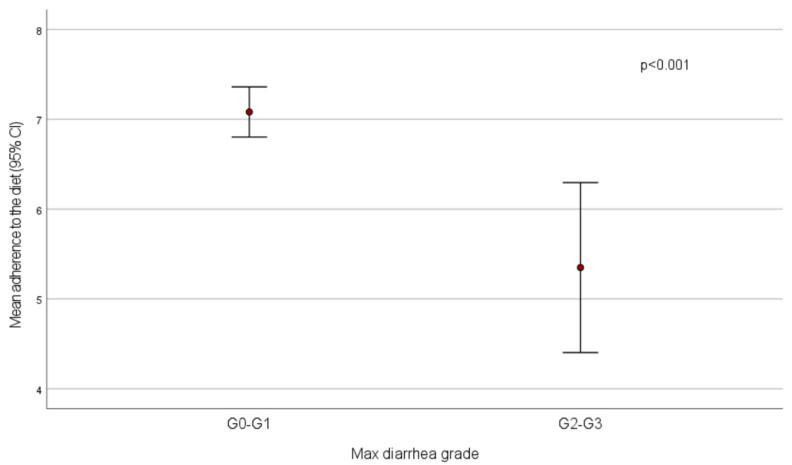
Mean adherence to the diet by diarrhea grade.

Mean adherence to the diet was significantly lower in patients who experienced diarrhea with maximum grade 2–3 compared to those who had no diarrhea or grade 1 diarrhea (score = 5.4 ± 1.9 vs. 7.1 ± 1.5, *p* < 0.001) (Figure 1). All patients who had high adherence had no diarrhea (*n* = 20) or grade 1 (*n* = 16) diarrhea, while low adherence was recorded in 3/77 (3.8%) patients without diarrhea and in 4/18 (22.2%) subjects with diarrhea reaching a maximum grade 2–3 (*p* < 0.001).

The mean adherence to the diet was found to be significantly lower in patients who experienced grade 2–3 diarrhea in comparison with those who had no diarrhea or grade 1 diarrhea in each of the first three chemotherapy cycles: first cycle, 4.6 vs. 7, *p* < 0.001; second cycle, 4.8 vs. 7, *p* = 0.004; third cycle, 4.7 vs. 7.4, *p* = 0.01.

Vitamin D blood levels at baseline were sufficient in 6.6% of patients, insufficient in 41.6%, and deficient in 25.5%. Vitamin D levels were significantly lower in patients who experienced grade 2–3 diarrhea during chemotherapy than those who had no diarrhea or grade 1 diarrhea (11.7 ± 9 vs. 15.9 ± 9, *p* = 0.03).

On the contrary, the mean maximum diarrhea severity was not significantly different according to the presence or absence of the primary tumor and liver or lung metastases.

**Figure 2:**
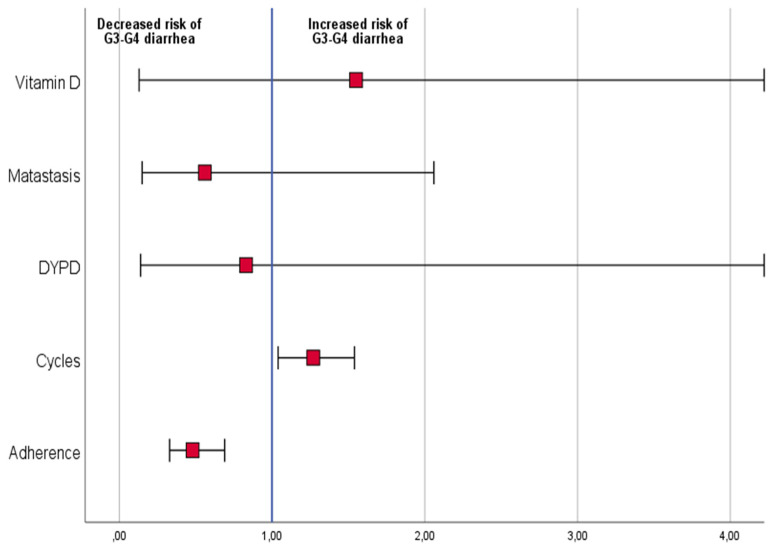
Risk of grade 3–4 diarrhea by vitamin D level at baseline, presence of metastases, DYPD mutation, number of chemotherapy cycles, and adherence to the study diet.

Logistic regression analysis, including dependent variables low vs. high–medium adherence to diet, *DYPD* mutation, presence vs. absence of metastases, Vitamin D low vs. high blood level, and the number of completed chemotherapy cycles, was performed. Patients with higher adherence to the diet were found to have a lower risk of grade 2 or 3 diarrhea (odds ratio: 0.5 (95% CI: 0.3–0.7, *p* < 0.001)). In addition, patients who completed a higher number of chemotherapy cycles had an increased risk of grade 2 or 3 diarrhea (odds ratio: 1.2 (95% CI: 1.0–1.5, *p* = 0.02)) (Figure 2).

## 4. Discussion

This prospective interventional monocentric study was performed to evaluate the efficacy of a nutritional regimen in reducing the incidence of chemotherapy-induced diarrhea to ≤10% in patients with metastatic colorectal cancer. This objective was met as medium-high adherence to the diet was associated with a low risk of grade 3 diarrhea (*p* < 0.001). Confounding factors, such as disease burden, cancer site, and *DYPD* mutations, were excluded.

These results are clinically important as severe diarrhea may impair the chemotherapy course and induce a relevant loss of nutrients and electrolytes. These effects may finally limit the efficacy and tolerability of chemotherapy and impact oncological outcomes.

A reduced incidence of diarrhea may facilitate the adherence to treatments and the regular completion of chemotherapy without dose delay and dose reduction, which may improve oncological outcomes. In addition, if the incidence of diarrhea is low, patients may have a better quality of life during chemotherapy. Indeed, all patients who received our diet completed the chemotherapy course without interruptions or dose changes.

The diet designed for this study was based on several needs: it matched the WCRF cancer prevention recommendations, excluded the risk of lactose intolerance, and reduced stool production by limiting the type and amount of vegetables and fruits.

We decided to match the WCRF recommendations to provide a regimen to be adopted during the chemotherapy time period and educate patients on a new lifestyle to be adopted after treatments for colorectal cancer. Cancer prevention recommendations include a high consumption of vegetables and a reduced amount of meat, which means adopting a Mediterranean diet instead of a western diet. Such a regimen is designed to restore gut microbial homeostasis. Indeed, gut microbiota are dynamic factors impacting the pathogenesis of colorectal cancer, the metabolism of drugs, and modulating responses to radiotherapy and immunotherapy [32,33]. Colorectal cancer survivors should be educated to adopt a safe lifestyle, as it has been observed that a diet rich in processed foods, animal fats, and red meat may change microbiota composition and increase the risk of colorectal cancer [34].

The blood level of Vitamin D at baseline was associated with an increased risk of diarrhea. The correction of Vitamin D levels should be considered in these patients, with a double aim: the prevention of diarrhea and reduction in cancer risk. Indeed, Vitamin D deficiency 1 year after surgical resection was associated with increased colon cancer relapse [35].

As expected, the number of chemotherapy cycles correlated with diarrhea incidence, which underpins the importance of a prolonged adherence to a safe diet. As our results identified the low adherence to the diet and the high number of chemotherapy cycles as risk factors for grade 3–4 chemotherapy-induced diarrhea, further investigation could validate them as predictor factors.

We acknowledge that the study has some limitations. This is a preliminary study and it has no control arm; our data are compared with historical data from the literature. All patients were recruited in one clinical center, and the study was monocentric; however, we obtained a large sample with the advantage of homogeneity in treatments. Patients were enrolled for a longer period than programmed (18 months) due to organization and the pandemic outbreak. It is also possible that behavior and environmental changes were present during the study. Notwithstanding these limitations, a large group of patients was included, and statistical analysis of the results was performed. This interventional study proposes the introduction of a new clinical practice, and univariate regression confirms the relevance of the diet to reduce the risk of diarrhea. Further large studies, with a randomized design, are necessary to confirm our findings.

## 5. Conclusions

In conclusion, a diet based on vegetables with a controlled fiber content (MMHD) is useful to control the incidence of chemotherapy-induced diarrhea. Such a diet may help patients adhere to chemotherapy and adopt a safe lifestyle aiming to reduce the risk of cancer relapse, even in survivor patients. Moreover, our data showed a relationship between Vitamin D levels and the incidence of diarrhea, suggesting a supplementation in patients with Vitamin D deficiency.

## Figures and Tables

**Table 1:** Demographic and clinical characteristics at baseline.

Demography	*n* = 137, *n* (%) or Mean (±SD)
Sex:	
Male	63 (46%)
Female	74 (54%)
Age (years)	65.3 (±11.4)
Weight (g)	66.7 (±14.7)
BMI (kg/m^2^)	24.6 (±4.8)
Weight status:	
Underweight (BMI < 18.5)	7 (5.1%)
Normal (BMI 18.5–25)	77 (56.2%)
Overweight (BMI 25–30)	39 (28.5%)
Obese (BMI > 30)	14 (10.2%)
Primary tumor site:	
Right	39 (28.5%)
Left	98 (71.5%)
Surgery	115 (83.9%)
Ostomy	28 (20.4%)
Peritoneal carcinosis	21 (15.3%)
Metastasis:	
Hepatic	80 (58.4%)
Pulmonary	55 (40.1%)
Bones	1 (0.7%)
Brain	2 (1.5%)
*DYPD* polymorphism:	19 (13.9%)
AG	5 (3.6%)
AT	3 (2.2%)
CG	2 (1.5%)
GA	8 (5.8%)
TC	1 (0.7%)
Toxicity risk (based on heterozygote or homozygote PDP variant):	
Increased	19 (13.9%)
Normal	73 (53.3%)
Not known	45 (32.8%)

## Data Availability

Data are available from the corresponding author upon reasonable request.

## Supplementary Materials

The following supporting information can be downloaded at: https://www.mdpi.com/article/10.3390/nu14091801/s1.

## References

[1] Escalante J., McQuade R.M., Stojanovska V., Nurgali K. (2017). Impact of chemotherapy on gastrointestinal functions and the enteric nervous system. Maturitas.

[2] Miroddi M., Sterrantino C., Simonelli I., Ciminata G., Phillips R.S., Calapai G. (2015). Risk of grade 3-4 diarrhea and mucositis in colorectal cancer patients receiving anti-EGFR monoclonal antibodies regimens: A meta-analysis of 18 randomized controlled clinical trials. Crit. Rev. Oncol. Hematol..

[3] Douillard J.Y., Cunningham D., Roth A.D., Navarro M., James R.D., Karasek P., Jandik P., Iveson T., Carmichael J., Alakl M. (2000). Irinotecan combined with fluorouracil compared with fluorouracil alone as first-line treatment for metastatic colorectal cancer: A multicentre randomised trial. Lancet.

[4] Saltz L.B., Cox J.V., Blanke C., Rosen L.S., Fehrenbacher L., Moore M.J., Maroun J.A., Ackland S.P., Locker P.K., Pirotta N. (2000). Irinotecan plus fluorouracil and leucovorin for metastatic colorectal cancer. Irinotecan Study Group. N. Engl. J. Med..

[5] Tournigand C., André T., Achille E., Lledo G., Flesh M., Mery-Mignard D., Quinaux E., Couteau C., Buyse M., Ganem G. (2004). FOLFIRI followed by FOLFOX6 or the reverse sequence in advanced colorectal cancer: A randomized GERCOR study. J. Clin. Oncol..

[6] André T., Boni C., Mounedji-Boudiaf L., Navarro M., Tabernero J., Hickish T., Topham C., Zaninelli M., Clingan P., Bridgewater J. (2004). Multicenter International Study of Oxaliplatin/5-Fluorouracil/Leucovorin in the Adjuvant Treatment of Colon Cancer (MOSAIC) Investigators. Oxaliplatin, fluorouracil, and leucovorin as adjuvant treatment for colon cancer. N. Engl. J. Med..

[7] Schmoll H.J., Cartwright T., Tabernero J., Nowacki M.P., Figer A., Maroun J., Price T., Lim R., Van Cutsem E., Park Y.S. (2007). Phase III trial of capecitabine plus oxaliplatin as adjuvant therapy for stage III colon cancer: A planned safety analysis in 1,864 patients. J. Clin. Oncol..

[8] Aprile G., Rihawi K., De Carlo E., Sonis S.T. (2015). Treatment-related gastrointestinal toxicities and advanced colorectal or pancreatic cancer: A critical update. World J. Gastroenterol..

[9] Rutledge D.N., Engelking C. (1998). Cancer-related diarrhea: Selected findings of a national survey of oncology nurse experiences. Oncol. Nurs. Forum.

[10] Cleeland C.S., Mendoza T.R., Wang X.S., Chou C., Harle M.T., Morrissey M., Engstrom M.C. (2000). Assessing symptom distress in cancer patients: The M.D. Anderson Symptom Inventory. Cancer.

[11] Rothenberg M.L., Meropol N.J., Poplin E.A., Van Cutsem E., Wadler S. (2001). Mortality associated with irinotecan plus bolus fluorouracil/leucovorin: Summary findings of an independent panel. J. Clin. Oncol..

[12] Benson A.B., Ajani J.A., Catalano R.B., Engelking C., Kornblau S.M., Martenson J.A., McCallum R., Mitchell E.P., O’Dorisio T.M., Vokes E.E. (2004). Recommended guidelines for the treatment of cancer treatment-induced diarrhea. J. Clin. Oncol..

[13] Kemeny N. (1994). Current approaches to metastatic colorectal cancer. Semin Oncol..

[14] Nair S. (2010). Vitamin D deficiency and liver disease. Gastroenterol. Hepatol..

[15] Fink M. (2011). Vitamin D deficiency is a cofactor of chemotherapy-induced mucocutaneous toxicity and dysgeusia. J. Clin. Oncol..

[16] World Cancer Research Fund Cancer Prevention Recommendations. https://www.wcrf.org/diet-and-cancer/cancer-prevention-recommendations/.

[17] AIOM (2020). Guidelines for Colon Cancer. https://www.aiom.it/wp-content/uploads/2020/10/2020_LG_AIOM_Colon.pdf.

[18] Artale S., Barzaghi S., Grillo N., Maggi C., Lepori S., Butti C., Bovio A., Barbarini L., Colombo A., Zanlorenzi L. (2022). Role of diet in the management of carcinoid syndrome: Clinical recommendations for nutrition in patients with neuroendocrine tumors. Nutr. Cancer.

[19] DRV of Nutrients and Energy for Italian Population (LARN). Revision IV (2012). https://sinu.it/larn/.

[20] Castagnini C., Luceri C., Toti S., Bigagli E., Caderni G., Femia A.P., Giovannelli L., Lodovici M., Pitozzi V., Salvadori M. (2009). Reduction of colonic inflammation in HLA-B27 transgenic rats by feeding Marie Ménard apples, rich in polyphenols. Br. J. Nutr..

[21] Raymond J.L., Morrow K. (2020). Krause and Mahan’s Food & the Nutrition Care Process.

[22] Binetti P., Marcelli M., Baisi R. (2006). Manuale di Nutrizione Clinica e Scienze Dietetiche Applicate.

[23] Bayless T.M., Rosensweig N.S. (1966). A racial difference in incidence of lactase deficiency. A survey of milk intolerance and lactase deficiency in healthy adult males. JAMA.

[24] Storhaug C.L., Fosse S.K., Fadnes L.T. (2017). Country, regional, and global estimates for lactose malabsorption in adults: A systematic review and meta-analysis. Lancet Gastroenterol. Hepatol..

[25] Cherny N.I. (2008). Evaluation and management of treatment-related diarrhea in patients with advanced cancer: A review. J. Pain Symptom Manag..

[26] Rossanese A., Nguyen T.M.D., Castelli F., Rizzato D., Napoletano G. (2005). Guidelines for the prevention and treatment of trevellers’ diarrhea. G. Ital. Med. Trop..

[27] World Health Organization Diarrhoeal Disease. https://www.who.int/news-room/fact-sheets/detail/diarrhoeal-disease#:~:text=Diarrhoea%20is%20defined%20as%20the,is%20normal%20for%20the%20individual.

[28] Malnutrition Advisory Group (2003). A consistent and reliable tool for malnutrition screening. Nurs. Times.

[29] García Cabrera S., Herrera Fernández N., Rodríguez Hernández C., Nissensohn M., Román-Viñas B., Serra-Majem L. (2015). KIDMED test; prevalence of low adherence to the Mediterranean diet in children and young; a systematic review. Nutr. Hosp..

[30] Martínez-González M.Á., Corella D., Salas-Salvadó J., Ros E., Covas M.I., Fiol M., Wärnberg J., Arós F., Ruíz-Gutiérrez V., Lamuela-Raventós R.M. (2012). Cohort profile: Design and methods of the PREDIMED study. Int. J. Epidemiol..

[31] Serra-Majem L., Ribas L., García A., Pérez-Rodrigo C., Aranceta J. (2003). Nutrient adequacy and Mediterranean Diet in Spanish school children and adolescents. Eur. J. Clin. Nutr..

[32] Silva M., Brunner V., Tschurtschenthaler M. (2021). Microbiota and colorectal cancer: From gut to bedside. Front. Pharmacol..

[33] Fong W., Li Q., Yu J. (2020). Gut microbiota modulation: A novel strategy for prevention and treatment of colorectal cancer. Oncogene.

[34] Watson A.J., Collins P.D. (2011). Colon cancer: A civilization disorder. Dig. Dis..

[35] Kim J., Baek D.W., Baek J.H., Kang B.W., Song S.H., Kim H.J., Park S.Y., Park J.S., Choi G.S., Kim J.G. (2021). Clinical impact of postoperative vitamin D deficiency on the recurrence of colon cancer after curative surgical resection. Anticancer Res..

