# Supplementary material for: A Nutritional Approach for the Management of Chemotherapy-Induced Diarrhea in Patients with Colorectal Cancer
Source: Nutrients. 2022 Apr 26;14(9):1801. doi: 10.3390/nu14091801 (PMC9100930; doi:10.3390/nu14091801)
Supplement: Supplementary file 1 [file nutrients-14-01801-s001.zip › nutrients-1669796-supplementary.pdf]

Supplementary Materials: Daily diet

- **Light tea** (black and/or green)

  Its astringent power increases if it is diluted and theine-free to avoid its gut motility increasing effect. Moreover, tea is rich in polyphenols (catechins), such as epigallocatechin-3-gallate (EGCG), one of the most powerful nutritional molecules against tissue invasion and angiogenesis (involved in carcinogenesis) [18].

- **Greek light yogurt (0–2%)**

  Yogurt has the same nutritional and energetic property as milk, but it is more digestible thanks to its small and more soluble protein particles and the presence of digested lactose. Moreover, lactic acid and other lactose ferments metabolites seem to inhibit the pathogenic gut microbe population [18].

  In particular, we have chosen Greek light yogurt because it provides high biological value proteins and calcium (150 mg/100 ml), and it has a low sugar and lactose content (0.5 g/100 ml) [18].

- **Rusks, breadsticks, crackers, crumb-free white bread or toasts, rice, and pasta**

  These solid and dry foods consumed with small amounts of liquids are preferred because they are more effective in reducing gut transit [18].

- **Whole peeled rice cream**

  It reduces gut motility and helps obtain cancer control thanks to its lower glucose compared to industrial rice [18].

- **Potatoes**

  They have low fiber content and astringent power. They are rich in minerals, which helps recover diarrhea-induced salts depletion and replace vegetables' contribution. Moreover, they have carotenoids and vitamin A precursors to protect the gut mucous membrane thanks to their alkaline properties.

- **Sweet potatoes or "Yam"**

Proposed in order to satisfy sweet taste needs. It contains twice as much sugar as potatoes, less vitamin PP but more vitamin A, and less fiber with the same alkaline properties.

- **Vegetables**

  All vegetables with a fiber content <3 g/100 g of full rate are permitted to provide the minimum physiological fiber amount.

  We have excluded the following from the diet:

- Vegetables with >3 g/100 g fibre content (for a pre-emptive intent);

- Vegetables rich in insoluble fiber (they increase fecal weight and speed fecal passage through colon) [18];

- Vegetables containing fermentable oligosaccharides (they pass undigested through the stomach and upper intestine. In the lower intestine, they are fermented by gas-producing bacteria causing flatulence and gut motility) [18].

Therefore eggplant, cabbage, raw carrots, red radish, broccoli, Brussels sprouts, artichokes, turnip, beet, leek, onion, garlic, parsley, and chicory were excluded from the diet.

- **Mushrooms**

  Low-fiber vegetables can be included to improve diet variety. Moreover, they are protective against cancer risks and relapse, especially mushrooms, shiitake, maitake, and enokitake [18].

- **Parmesan cheese**

  Thanks to the long maturation, it is completely lactose-free, rich in calcium and sodium, and effective on water retention. It improves nutrition with high nutritional value without increasing meal quantities.

- **Meat and fish**

  The main sources of proteins with a high biological value are meat and fish. The consumption of red meat (beef, pork, and lamb meat) is minimized in favor of white meat (chicken, turkey, and rabbit) and fish

(omega-3 fatty acids); processed meat is excluded. Several studies show red and processed meat association with pro-oxidant effect against colic cells and the alteration of crypts [18], so we decided to respect WCRF guidelines to provide a secure amount and the best frequency of their consumption [18].

- **Peeled red lentils**

The lack of outer coating reduces the fiber quantity compared to other vegetables.

- **Eggs**

They have a suitable quantity of vitamin D (1.75 g) [18]. Furthermore, there is not enough evidence to advise against their consumption on the matter of cancer prevention or its relapses [18].

- **Soy tofu**

Soy contains a suitable quantity of proteins with a high biological value; thus, it is a perfect replacement for meat. The moderate consumption of foods naturally derived from soy may be scheduled within a balanced diet, but soy-based supplements should be excluded [18].

- **Lemon**

It is used as a flavoring if tolerated, as it is known to perform an astringent and bactericidal actions.

- **Raw extra virgin olive oil**

Used to flavor cooked foods: it is important for its energetic contribution and absorption of fat-soluble vitamins.

- **Apple**

Its tannin has an astringent and anti-inflammatory power [18].

- **Banana**

It has anti-diarrhoeal antioxidants as well as antibacterial activity [18]. It is responsible for gut mucosal regulation, gut microbial population balance, and colon function normalization.

- **Apricots, peaches, strawberries, and blueberries**

Chosen from <7 g sugar and <3 g fiber fruits to avoid the adverse intestinal effect. They must be peeled before consumption to avoid gut discomfort caused by their fiber.

Moreover, a small portion of jam is included. It should be consumed in association with rusks or other cereals to avoid gut osmotic effects.
